# Evaluation of the use of photobiomodulation following the placement of elastomeric separators: Protocol for a randomized controlled clinical trial

**DOI:** 10.1097/MD.0000000000017325

**Published:** 2019-10-25

**Authors:** Silvana Machado Ortega, Marcela Leticia Leal Gonçalves, Tamiris da Silva, Anna Carolina Ratto Tempestini Horliana, Lara Jansiski Motta, Olga Maria Altavista, Silvia Regina Olivan, Ana Eliza Castanho Garrini dos Santos, Ana Luiza Cabrera Martimbianco, Raquel Agnelli Mesquita-Ferrari, Kristianne Porta Santos Fernandes, Sandra Kalil Bussadori

**Affiliations:** a Postgraduate Program in Biophotonics Applied to Health Sciences; b Postgraduate Program in Rehabilitation Sciences, Universidade Nove de Julho, UNINOVE; c Postgraduate Program in Health and Environment, Universidade Metropolitana de Santos, UNIMES, Santos, SP, Brazil.

**Keywords:** cytokines, orthodontics, pain, photobiomodulation

## Abstract

**Background:**

Pain stemming from the placement of elastomeric separators and the exchanging of wires and accessories is the greatest reason for abandoning orthodontic treatment. Indeed, discomfort related to treatment exerts a negative impact on quality of life due to the difficulty chewing and biting. This paper proposes a study to evaluate the analgesic effects of photomiobodulation (PBM) on individuals undergoing orthodontic treatment.

**Methods:**

The sample will be composed of 72 individuals who receiving elastomeric separators on the mesial and distal faces of the maxillary first molars. The patients will be randomly allocated to 2 groups: an experimental group irradiated with low-level laser and a sham group submitted to simulated laser irradiation. Upon the placement of the separators, the experimental group will receive a single application of PBM on the mesial and distal cervical portion and apical third of the molars. Perceived pain will be analyzed after one hour using the visual analog scale in both groups. Samples will be taken of the gingival crevice with absorbent paper for 30 seconds for the analysis of cytokines using ELISA and the results of the 2 groups will be compared. The patients will sign a statement of informed consent. Statistical analysis will be performed with the Student's *t* test and analysis of variance (ANOVA).

**Discussion:**

The expectation is that the patients in the irradiated group will have a lower perception of pain and lower quantity of cytokines compared to those in the sham group. The purpose of the study is to establish an effective method for PBM with the use of low-level infrared laser (Ga-Al-As with a wavelength of 808 nm and output power of 100 mW) for reductions in pain and inflammatory cytokines related to orthodontic treatment.

**Trial registration:**

This protocol was registered in ClinicalTrial.gov, under number NCT03939988. It was first posted and last updated in May 6, 2019.

## 1 Introduction

Despite recent progress in the field, approximately 90% of orthodontic patients still associate treatment with pain, which is the most common reason for abandoning treatment.^[1,2]^ Pain caused by the placement of elastomeric separators at the onset of treatment is very common and intense in the first two days.^[3,4,5]^ Prostaglandins, substance P, encephalin, leukotrienes, bradykinins and histamines are the main substances responsible for this process due to their role in sensitizing nerve endings, aggravating inflammation and increasing pain.^[6,7,8]^ Cytokines are among the primary inflammatory mediators and constitute a group of low molecular weight proteins secreted by cells, such as the chemokine IL-8, which is responsible for leukocyte recruitment and is a potent chemoattractant for neutrophils, which explains its high concentration at tissue injury sites.^[9,10–12]^

New theories are being studied and photobiomodulation (PBM), especially low-level laser therapy (LLLT), has proven to be effective due to its therapeutic properties and lack of side effects. PBMis reported to achieve stimulation and a regenerative effect on the molecular level in injured tissue.^[13–17]^

This paper describes the protocol for a study with the purpose of evaluating PBM with a single session of infrared LLLT in individuals having received elastomeric separators at the onset of orthodontic treatment to determine its effects on reducing pain and the quantity of inflammatory cytokines.

## 2 Methods/design

### 2.1 Type of study

A randomized, controlled, clinical trial will be conducted following the Consolidated Standards of Reporting Trials (CONSORT statement). The study will involve patients recruited from the dentistry clinic of University Nove de Julho (São Paulo, Brazil).

### 2.2 Trial registration

The project for the proposed study received approval from the Human Research Ethics Committee of Universidade Nove de Julho (process number: 13694419.1.0000.5511). This protocol was registered in ClinicalTrial.gov, under number NCT03939988. It was first posted and last updated in May 6, 2019.

### 2.3 Calculation of sample size

The sample size for the evaluation of the analgesic effect of PBM was determined based on the results of previous studies by Qamruddin et al using the G∗Power software (version 3.1.9.2). Considering a 5% level of significance, 80% test power and effect size >.60 for the detection of differences between groups, a total of 36 individuals will be needed for each group.
 
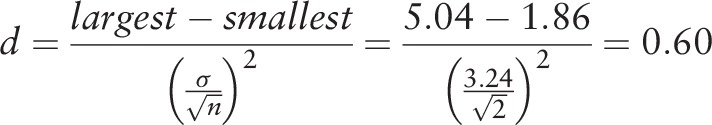


### 2.4 Recruitment and Randomization of patients

The sample will consist of 72 male and female individuals 12 years of age or older at the onset of orthodontic treatment. They will be recruited by invitation at the dentistry clinic of University Nove de Julho. As they will already be in treatment, we expect adherence to the protocol will be satisfactory and the sample size will be achieved without much difficulty. The patients will be randomly divided into an experimental group and sham group – each with 36 patients. The patients will be randomly allocated to either an experimental group (active laser irradiation) or sham group (simulated laser irradiation). Randomization will be performed by asking the volunteers to choose between 2 envelopes. One envelope will contain a piece of paper with the letter A (corresponding to active laser) and the other will contain a piece of paper with the letter B (corresponding to sham laser). The volunteers will not be aware of which treatment they are receiving. The patients who received the envelope with the letter A will be submitted to the placement of elastomeric separators, followed by infrared LLLT and those who received the envelope with the letter B will be submitted to the placement of elastomeric separators, followed by simulated LLLT.^[18]^

### 2.5 Inclusion criteria

Individuals at the onset of orthodontic treatment and who have not previously undergone treatment will be included in the study. We will evaluate clinical conditions through a previous examination with probing of the gingival crevice for the inclusion of only patients with a healthy periodontium, sound maxillary molars with interproximal contacts between the second molar and premolar in the permanent dentition phase having not made use of anti-inflammatory drugs or analgesics in the previous 4 days.^[18]^

### 2.6 Exclusion criteria

Patients with systemic diseases who habitually take medications and those with periodontal disease will be excluded from the study.^[18,19]^

### 2.7 Operational plan

**Figure 1:**
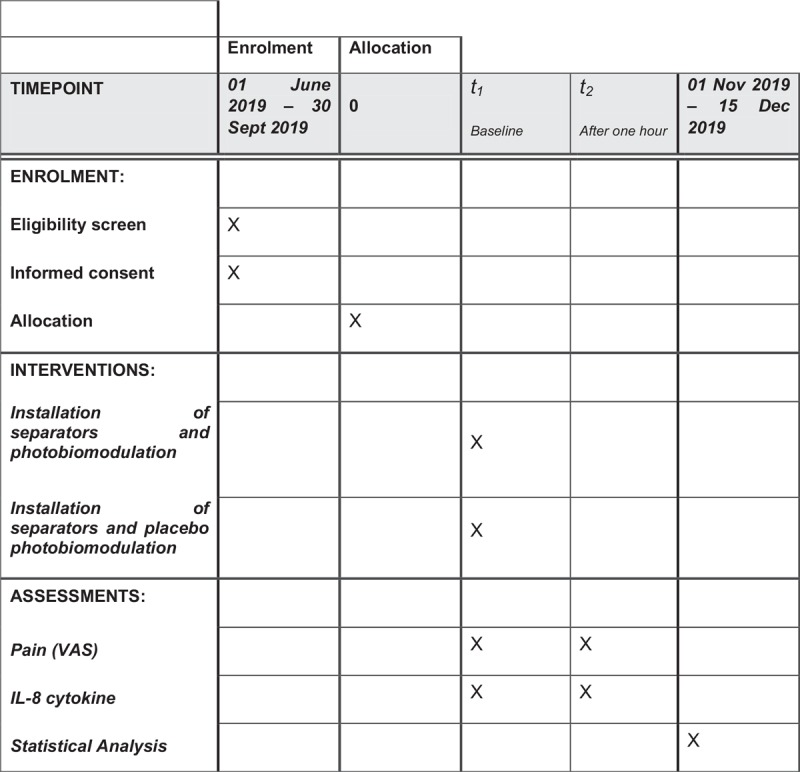
SPIRIT figure as recommended by 2013 SPIRIT Statement.

The protocol is in accordance with the 2013 SPIRIT (Standard Protocol Items: Recommendations for Interventional Trials) Statement. The SPIRIT checklist can be found as an additional file and Figure 1 is the SPIRIT figure.

After the researcher clarifies the objectives of the study, the participants will sign a statement of informed consent. The patients will be randomly divided into 2 groups using envelopes containing either the letter A (experimental group – active laser irradiation) or the letter B (sham group – simulated laser irradiation). The participants will have no knowledge of the treatment (active or sham) to which they will be submitted. The experimental group will be irradiated with infrared LLLT and the sham group will be submitted to the simulated procedure, during which the device will emit sound, but no radiant energy will be produced. Elastomeric separators will be placed with the assistance of dental floss between the mesial and distal interproximal contact of the permanent maxillary first molars, followed by immediate irradiation (active or sham). The procedure will be performed in an isolated room with both the researcher and patient wearing protective eyewear.^[18]^

Immediately after the placement of the separators, a single session of LLLT will be performed with 3 vestibular and 3 lingual applications to the maxillary first molars at three points of the interproximal papillae (cervical, mesial, and distal thirds) as well as the apical third of the roots.^[20]^ Fluid will be collected from the gingival crevice in both groups during irradiation and 1 hour after irradiation for subsequent analysis.^[18]^

### 2.8 Application of low-level laser

A Ga-Al-As laser (Therapy XT, DMC Equipamentos, São Carlos, Brazil) will be used with an output power of 100 mW. The tip of this device has a diameter of 600 μm and 2 optic fibers – one for delivering a wavelength of 808 nm (infrared) and another for delivering a wavelength of 660 nm (red). In the proposed study, the individuals in the experimental group will receive infrared laser in continuous mode at a wavelength of 808 nm.

The tip of the laser device will be positioned perpendicularly to the mucosa without exerting pressure. In the experimental group, each point will be irradiated with 2 J for 20 seconds, totaling 12J per tooth (6 J on the vestibular side and 6 J on the lingual side). Table 1 lists the dosimetric parameters, which were selected based on previous studies in the literature. One hour after the placement of the separators, the patients will report their perception of pain by marking their level of discomfort on the visual analog scale (VAS) ranging from 0 (absence of pain) to 10 (intolerable pain). The patients will be instructed not to make use of medications during the study ^[21]^.

**Table 1:**
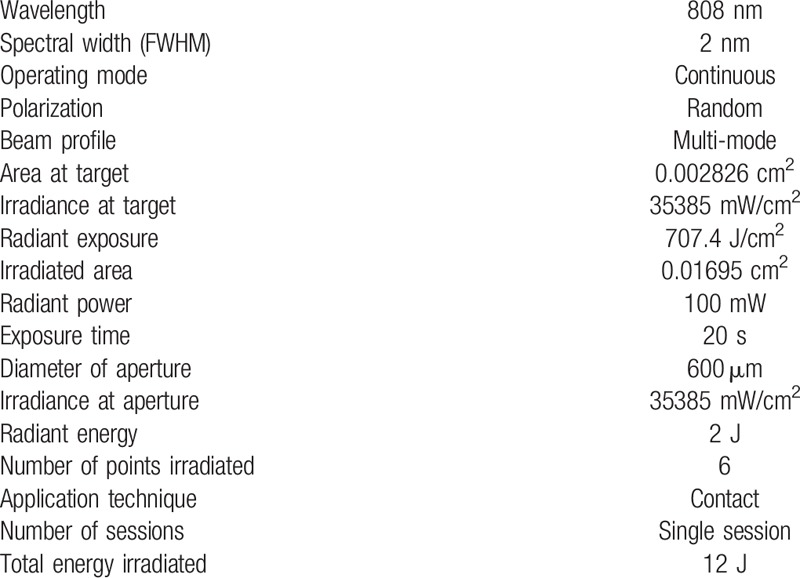
Dosimetric parameters.

### 2.9 Collection of gingival crevicular fluid

Gingival crevicular fluid will be sampled in both groups for the evaluation of cytokines. Before and 1 hour after the placement of the separators, absorbent paper cones will be carefully inserted into the cervical middle third of the vestibular face of each tooth until encountering mild resistance and maintained in position for 30 seconds. Prior to the insertion of the paper cones, supragingival plaque will be delicately removed from the tooth surface. For such, the teeth will be isolated with cotton rolls and dried with compressed air. The volume of the gingival crevicular fluid will be calculated by the difference in weight of the paper cone before and after sampling at a proportion of 1 g/ml. The samples will be collected separately in sterile tubes containing 2 ml of phosphate buffer solution (pH 7.4)and stored at −70° C until the analysis of the cytokines IL-1β, IL-6, IL-8, IL-10, and TNF-α.^[22]^ Cytokine levels will be determined by a single examiner using ELISA interleukin kits, strictly following the manufacturer's instructions.^[22–25]^

### 2.10 Analysis of results

The data will be tabulated and treated using the SPSS 22 program. Qualitative data will be expressed as frequency or percentage. Quantitative data will be expressed as mean and standard deviation values. The Shapiro-Wilks and Kruskal-Wallis tests will be used to determine the normality of the data. Associations between variables will be tested by comparing means of the VAS and ELISA results with the Student's *t* test and analysis of variance (ANOVA). The level of significance for all tests will be set to 5% (*P* < .05).

## 3 Discussion

The expected result will be the certification that infrared laser has an analgesic effect and reduces the quantity of inflammatory cytokines in the gingival crevicular fluid of patients at the onset of orthodontic treatment. The expected benefits for the volunteers will be greater comfort, with a reduction in pain stemming from the placement of elastomeric separators.

## Author contributions

**Conceptualization:** Silvana Machado Ortega, Raquel Agnelli Mesquita-Ferrari, Kristianne Porta Santos Fernandes, Sandra Kalil Bussadori.

**Data curation:** Ana Luiza Cabrera Martimbianco.

**Formal analysis:** Anna Carolina Ratto Tempestini Horliana, Lara Jansiski Motta.

**Investigation:** Silvana Machado Ortega, Tamiris da Silva, Olga Maria Altavista, Silvia Regina Olivan, Ana Eliza Castanho Garrini dos Santos, Ana Luiza Cabrera Martimbianco.

**Methodology:** Silvana Machado Ortega, Tamiris da Silva, Olga Maria Altavista, Silvia Regina Olivan, Ana Eliza Castanho Garrini dos Santos, Ana Luiza Cabrera Martimbianco.

**Software:** Lara Jansiski Motta.

**Supervision:** Anna Carolina Ratto Tempestini Horliana, Lara Jansiski Motta, Raquel Agnelli Mesquita-Ferrari, Kristianne Porta Santos Fernandes, Sandra Kalil Bussadori.

**Visualization:** Anna Carolina Ratto Tempestini Horliana.

**Writing – original draft:** Silvana Machado Ortega, Tamiris da Silva.

**Writing – review & editing:** Marcela Leticia Leal Gonçalves, Sandra Kalil Bussadori.
